# Survey on Tuberculosis Patients in Rural Areas in China: Tracing the Role of Stigma in Psychological Distress

**DOI:** 10.3390/ijerph14101171

**Published:** 2017-10-04

**Authors:** Minlan Xu, Urban Markström, Juncheng Lyu, Lingzhong Xu

**Affiliations:** 1 School of Public Health, Shandong University, Wenhuaxi Road 44, Jinan 250012, China; Lzxu@sdu.edu.cn; 2 Department of Social Work, Umeå University, 90187 Umeå, Sweden; urban.markstrom@umu.se; 3 Department of Public Health, Weifang Medical University, Weifang 261000, China; cheng_china@163.com

**Keywords:** stigma, psychological distress, K10, scale, tuberculosis

## Abstract

Depressed patients had risks of non-adherence to medication, which brought a big challenge for the control of tuberculosis (TB). The stigma associated with TB may be the reason for distress. This study aimed to assess the psychological distress among TB patients living in rural areas in China and to further explore the relation of experienced stigma to distress. This study was a cross-sectional study with multi-stage randomized sampling for recruiting TB patients. Data was collected by the use of interviewer-led questionnaires. A total of 342 eligible and accessible TB patients being treated at home were included in the survey. Psychological distress was measured using the Kessler Psychological Distress Scale (K10). Experienced stigma was measured using a developed nine-item stigma questionnaire. Univariate analysis and multiple logistic regression were used to analyze the variables related to distress, respectively. Odds ratios (ORs) and 95% confidence intervals (CIs) were used to present the strength of the associations. Finally, the prediction of logistic model was assessed in form of the Receiver Operating Characteristic (ROC) curve and the area under the ROC curve (AUC). According to the referred cut-off point from K10, this study revealed that 65.2% (223/342) of the participants were categorized as having psychological distress. Both the stigma questionnaire and the K10 were proven to be reliable and valid in measurement. Further analysis found that experienced stigma and illness severity were significant variables to psychological distress in the model of logistic regression. The model was assessed well in predicting distress by use of experienced stigma and illness severity in form of ROC and AUC. Rural TB patients had a high prevalence of psychological distress. Experience of stigma played a significant role in psychological distress. To move the barrier of stigma from the surroundings could be a good strategy in reducing distress for the patients and TB controlling for public health management.

## 1. Introduction

Tuberculosis (TB) is a chronic infectious disease that influences not only physical health, but also the psychological and social well-being of patients. Psychological co-morbidity prolongs the course of illness, involves lengthier diagnostic procedures, increases treatment costs, and reduces treatment efficacy [1,2,3]. A meta-analysis study showed that depressed patients could have a three-fold higher risk of being non-adherent to treatment than non-depressed patients [4], which non-adherence to medication is a big challenge to tuberculosis (TB) controlling. Therefore, it is of great relevance that the psychological health of TB patients is studied.

A Pakistani study found that up to 46% of TB patients had depression [5]. Several other studies showed that TB patients suffered more co-morbid mental disorders than the general population because of the length of treatment, loss of income, stigma, and isolation from society [6,7]. We therefore assumed that TB patients in China suffer a high prevalence of psychological distress as well.

With regard to the studies about different variables related to distress in TB patients from different regions, some discovered that gender [8,9], age, and socio-economic status were related to psychological distress [10], while others found that illness severity was closely related to depression in TB patients [5,10]. Although there were a lot of studies about the role of stigma in mental health, particular in the fields of psychology, and Human Immunodeficiency Virus and Acquired Immune Deficiency Syndrome (HIV/AIDS) [11]. However, there were few studies on the role of stigma in distress among TB patients [12,13,14].

Stigma has been long studied by researchers, among whom Goffman’s work is more concerned with the stigmatizing consequences on the individual. According to this, when a disease label is attached to a patient, the label could have the power to spoil the sufferer’s identity [15]. As to the term of stigma, there are different definitions [16], which depend on the study perspective. These include the conceptualization of mental illness stigma. Link’s research focused on the subjective expectation and experience of being discriminated [17]; while Corrigan’s study focused on cognitive and behavioral characteristics [18]. For the TB patients in this study, we defined stigma as the individual experience or perceptions of attitudes and ways that they were treated by the surrounding people in daily life during the illness.

China is still among the countries with the highest burden of TB, despite the great progress that has been made in controlling TB over the past decades [19]. In China, TB is thought of as a “dirty disease”, and there is an estimate that 80% of registered TB patients live in rural areas [20], where more and more residents have knowledge of TB and its contagiousness. Public stigma against TB patients is due to the fears of being infected, and thus TB patients may hide their infectious conditions. These situations can not only bring distress to patients, but also interfere with early detection, treatment, and recovery for TB patients [21]. Therefore, in this study we aim to (1) investigate psychological distress among the rural TB patients, and further (2) to trace the role of experienced stigma in distress in the case of rural TB patients. The results may provide healthcare professionals with the evidence for supporting future mental health intervention strategies related to TB stigma and improvements in the control of TB.

## 2. Methods

### 2.1. Study Population

This study was part of a survey on rural TB patients in Shandong Province, which is located in eastern China with a population of nearly 100 million and unbalanced economic development: poor in the west and rich in the east. First, three regions based on the economic level were sampled by using multi-stage random sampling. Then, two counties in each sampled region were selected, and six rural towns in each sampled county were selected randomly. Therefore, 36 towns in the six sampled counties were chosen as the survey sites. In the end, patients were cluster-sampled from the 36 selected towns, where all the eligible TB patients were included as samples for the study. The sampling method has been described previously in another paper [22]. In China, county TB dispensaries (CTD) are the official institutions responsible for the management of TB cases in rural areas. In this study, complicated cases who may suffer extra psychological burden such as hospitalized patients, recurrent cases after failed treatment, drug-resistant cases, and TB patients with other chronic complications, were excluded. Next, TB patients being treated at home were recruited, and household visits were appointed through telephone calls with the help of local CTD staff. Then, interviewer-led questionnaires were used in the structured face-to-face interviews, with each interview lasting approximately one hour. For quality control, the interviewers, consisting of institute staff and graduate students, attended a training workshop to study the survey protocol [22]. At the same time, the CTD staff helped with supervision in the field survey.

### 2.2. Questionnaire and Types of Variables

The developed questionnaire consisted of sociodemographic characteristics, medical conditions, questions to measure experienced stigma, and a scale to measure psychological conditions. Sociodemographic characteristics included age, gender, living with a partner (yes/no), school years, and self-reported household income (high/middle/low) in comparison to local level, while medical conditions were presented in the form of treatment duration, and self-rated illness severity (no/minor/moderate/severe). Based on the mean values, continuous variables: age, school years, and treatment duration were further dichotomized into binary variables: age groups (old/young), education levels (high/low), and treatment length (long/short), in case variables in the original format or in multi-categorized format showed no statistical significance in models.

### 2.3. Measures of Experienced Stigma

In this study, experienced stigma was conceptualized as individual experiences from treatment by family members, neighbors, and friends in daily life since the diagnosis was confirmed. Based on the item-response theory in identifying items that made maximal discrimination of respondents and literatures’ reviews about stigma scales [23,24,25], the nine-item stigma questionnaire was developed, taking into consideration the social and cultural backgrounds that exist in China (Appendix). The items were rated on the four-point Likert scale ranging from 1 to 4 (strongly disagree/disagree/agree/strongly agree), respectively. The obtained total scores ranged from 9 to 36. The reliability and validity of the scale were already validated in another study [26]. As comparison, stigma levels were categorized according to the same definition. High stigma level was defined if the summed score was more than the median score [26].

For the stigma questionnaire in this study, the total Cronbach’s α was 0.91, and the corrected item-total correlations were from 0.57 to 0.72, which indicated good reliability. Factor analysis extracted one factor with eigenvalue 5.14, which could explain 57.15% of the variance. The factor loadings ranged from 0.65 to 0.79.

### 2.4. Measures of Psychological Distress

The five-point Likert Kessler Psychological Distress Scale (K10) (Chinese version), which has 10-item questions measuring anxiety or depression and other psychological-related symptoms in the past four weeks [27], was used to measure psychological status in this study. Patients with K10 scores ≥16 were defined as having psychological distress according to the cut-off point 16, which showed agreeable sensitivity and specificity in detecting psychological distress [10]. The use of K10 as a measuring instrument for psychological health surveys has become popular in the past 10 years [28,29,30]. The Chinese version of K10 showed a good consistency with the Symptom Checklist-90 (SCL-90) [31].

In this study, the total Cronbach’s α for K10 was 0.93 and the corrected item-total correlations varied from 0.61 to 0.81, which showed that the internal consistency of the scale was good. Further factor analysis extracted one factor with eigenvalue 6.06, which could explain 60.85% of the variance. The factor loadings ranged from 0.68 to 0.86.

The psychometric properties of both scales showed good internal consistency, and the unidemensional constructed validity in measurement.

### 2.5. Data analysis

After data collection, a database was set up using EpiData 3.1 (EpiData Association, Odense, Denmark). First, the level of psychological distress in accordance with the K10 definition was presented by sociodemographic characteristics, clinical conditions and experienced stigma. Second, univariate analysis was used to test the associations between the variables and psychological distress by adopting Pearson’s Chi-Square. Third, variables with *p* value less than 0.05 from the univariate analysis were further analyzed in the multiple logistic regression models. Odds ratios (ORs) and 95% confidence intervals (CIs) were used to present the strengths of the associations. Finally, the predictive capability of the logistic model was assessed adopting the Receiver Operating Characteristic (ROC) curve and the area under the ROC curve (AUC). The statistical significance *p* value was set at *p* < 0.05. Statistical Analysis System (SAS 9.2 version) software (SAS Institute Inc., Cary, NC, USA) was used in data analysis.

## 3. Results

### 3.1. Sample Characteristics and psyChological Distress by Variables

In accordance with the sampling criteria, there were 372 patients in the sample frame, and among them, 342 tuberculosis eligible patients were included in this survey. Of these rural TB patients, 70.47% (241/342) of the surveyed were men. The average age was 53.57, ranging from 15 to 96, and 42.11% patients were above 60 years old. The patients had an average of 5.70 years of school education. With regard to marital status, 78.07% (267/342) of patients lived with a partner or spouse. 37.13% (127/342) of the patients ranked their household income to be below the local average level. The mean length of treatment was 3.11 months; while 73.68% (252/342) patients rated their illness severity as moderate or severe (Table 1). Moreover, the average score of stigma was 23.6, ranging from 9 to 39, while the median score was 24.

For the patients, the average score of K10 was 20.25 ranging from 10 to 47, while the standard deviation was 8.22 among the patients. According to the K10 definition, 65.20% (223/342) of the surveyed patients were categorized as having psychological distress. Women, older patients, patients with less schooling, and patients with a lower household income had higher percentages of psychological distress. However, further univariate analysis using the Chi-Square test showed that only school education and household income were statistical significant variables (*p* < 0.05) in relation to psychological distress. Meanwhile, marital status seemed to have no association with distress in this study (Table 1).

The Chi-Square test on the clinical variables found that illness severity was a significant variable (*p* < 0.0001) to psychological distress, while the treatment length (*p* > 0.05) was not. As for experienced stigma, 45.32% (155/342) patients had a high level of experienced stigma, and experienced stigma was found to be significantly associated with psychological distress as well, according to the Chi-Square test (*p* < 0.0001). In summary, univariate analysis revealed that school years, household income, illness severity, and experienced stigma were variables with statistical significance *p* < 0.05 (Table 1), and these were further analyzed in the following multiple logistic regression.

### 3.2. Variables to Psychological Distress in the Logistic Regression Analysis

After checking for absence of multicollinearity and interactions, significant variables produced by the univariate analysis given above were further adjusted in the logistic regression model. It was found that self-rated illness severity: moderate (OR = 2.40, 95% CI: 1.38–4.20) or severe (OR = 6.59, 95% CI: 3.43–12.69) in contrast to no or minor symptoms, and a high level of experienced stigma (OR=2.24, 95% CI: 1.36–3.67) compared to low level of experienced stigma (Table 2), were significant variables for psychological distress. However, school years and household income were no longer significant (*p* > 0.05) in the multiple logistic model (Table 2).

### 3.3. Predictive Capability of the Multiple Logistic Regression Model

**Figure 1:**
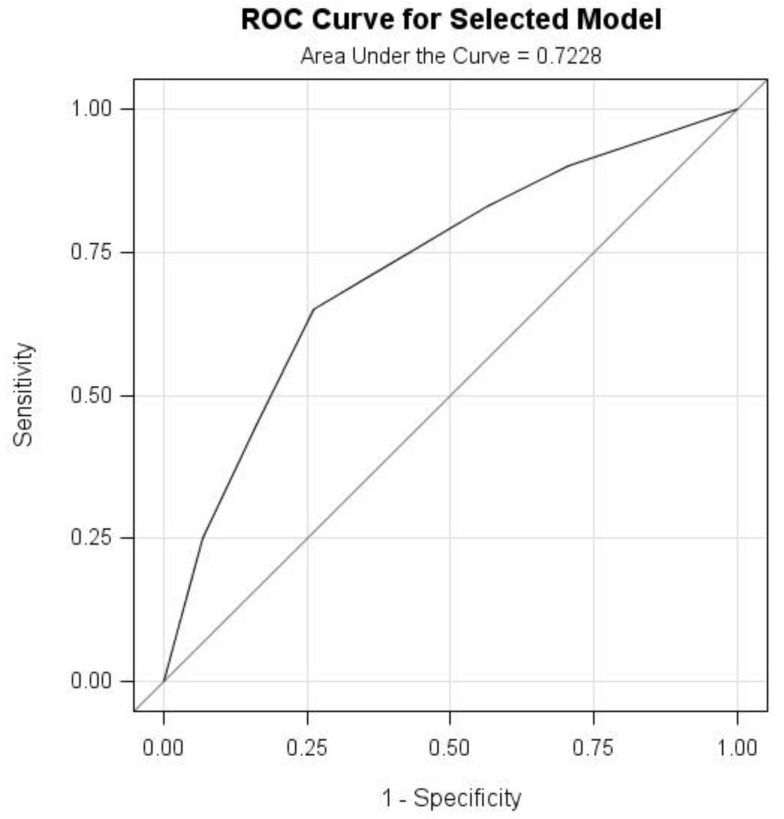
Receiver Operating Characteristic (ROC) curve and Area under the Curve (AUC) for the logistic regression model.

The ROC curve is a plot of sensitivity (distress predicted to be distress) versus (1-specificity) (non-distress predicted to be distress), which could be used to measure and visualize the predictive capability of the logistic regression model by using experienced stigma and illness severity. Meanwhile, the area under the ROC curve (AUC) could be used as a model measure of goodness of fit. Figure 1 presented that AUC was 0.7228 for the fitted model, which was good.

## 4. Discussion

Having TB could impose a psychological burden on patients. In this study, TB patients in rural areas showed a high percentage of psychological distress by using K10, which could not be ignored. This study also found that experienced stigma was significantly associated with psychological distress among rural TB patients, and so was illness severity. The K10 and stigma questionnaire proved to have good reliability and validity. Both experienced stigma and illness severity could well predict psychological distress among TB patients in the logistic model.

It is quite understandable that the degree of illness severity could make patients worried and frustrated, and this could result in distress. One study found that illness perception could predict depression in patients with heart disease [32]. These findings proved to be consistent with other studies as well [5,10].

There has been a scarcity of TB stigma assessment in quantitative methods [21], especially with the role of stigma in psychological distress of TB patients. In China, one study found that TB stigma even existed among health care providers against patients [33]. In our study, for the first time we further explored the role of experienced stigma in psychological distress among TB patients in rural communities, which focused on individual experiences from attitudes and behaviors of the surrounding neighbors, friends, and relatives. Such experience could be interpreted as the negative effects of diagnostic labels on the TB patients [17]. Once being diagnosed with TB, patients with the label could experience various stigma from the surroundings in daily life. Furthermore, they may respond to these attitudes or behaviors by isolating themselves [34], which would then further block social communication and successful TB control. However, as a consequence of TB stigma [13,14,34,35], distress of individuals was less frequently mentioned. Our findings presented that the experienced stigma played an important role in distress among the TB patients. The results not only explained the association between stigma and distress, but also could contribute to TB control strategies through reduction of potential negative effects such as delayed diagnosis or poor treatment adherence [9,35,36,37].

There is some evidence that gender, age, household income, and school years are significant variables to mental health in populations living in certain social settings, or coming from certain cultural backgrounds [37,38,39], but this was not the case for this study, as had been supposed in the multivariate logistic model. This may partly be attributed to the policy of providing essential drugs for TB free of charge, a recent policy which may have eased the economic-related psychological burden on patients to some degree, and may increase life satisfaction for patients. Additionally, it could be said that education and household income played a role on distress but not strong enough to reach statistical significance or a precise estimation when controlling for other independent variables in the multivariate models. Thus, more evidence is needed.

Each study has its weaknesses, and for this study, this was cross-sectional, as related variables could not be inferred to as being causal, and would need further observation in a longitudinal study. Second, this study was conducted in rural areas, and findings could be explored for urban inhabitants or migrants in the cities. Moreover, the effects of stigma on distress may be underestimated among the complicated cases who were excluded from the study. Finally, other variables such as self-esteem may have mediating effects in mental health, and are worth further exploring in future studies.

So far, China has conducted many health care measures to help TB patients, and this has contributed to the success of TB control. According to the World Health Organization (WHO) definition of health, there is no state of health that does not include mental health, implying that health must include psychological health. This is the same for the psychological health of TB patients. This study contributed to the characterization of the relation between stigma and psychological distress among rural TB patients, and this was the first exploration in this field in China.

## 5. Conclusions

Rural TB patients presented a high level of psychological distress; both K10 and the stigma questionnaire proved to be good tools for measurements. Experienced stigma was significantly associated with psychological distress, which implied the necessities of reducing TB related stigma through intervention studies about attitude or behavior changes in public, and self-efficiency in patients.

## Figures and Tables

**Table 1:** Psychological distress by sociodemographic variables, clinical conditions and experienced stigma.

Variables	Total (*N* = 342)	Psychological Distress		Chi-Square Test *p* Value
	*n* (%)	PD *n* (%)	Non-PD *n* (%)	
Gender				0.20
Men	241 (70.47)	152 (63.07)	89 (36.93)	
Women	101 (29.53)	71 (70.30)	30 (29.70)	
Age groups				0.56
Old	197 (57.60)	131 (66.50)	66 (33.50)	
Young	145 (42.40)	92 (63.45)	53 (36.55)	
Education levels				0.04
High	181 (52.92)	109 (60.22)	72 (39.78)	
Low	161 (47.08)	114 (70.81)	47 (29.19)	
Marital status				0.43
With partner	267 (78.07)	177 (66.29)	90 (33.71)	
Without partner	75 (21.93)	46 (61.33)	29 (38.67)	
Household income				0.009
Average or above	215 (62.87)	129 (60.00)	86 (40.00)	
Below average	127 (37.13)	94 (74.02)	33 (25.98)	
Treatment length				0.34
Short	103 (30.12)	71 (68.93)	32 (31.07)	
Long	239 (69.88)	152 (63.60)	87 (36.40)	
Illness severity				<0.0001
No/minor	90 (26.32)	38 (42.22)	52 (57.78)	
moderate	134 (39.18)	86 (64.18)	48 (35.82)	
Severe	118 (34.50)	99 (83.90)	19 (16.10)	
Experienced stigma				0.0001
Low	187 (54.68)	105 (56.15)	82 (43.85)	
High	155 (45.32)	118 (76.13)	37 (23.87)	

PD: psychological distress.

**Table 2:** Analysis of variables to psychological distress in multiple logistic regression model.

Variables	Psychological Distress		
	Adjusted OR	95% CI	*p* Value
Education levels			
High	1.0		
Low	1.22	0.73–2.02	0.45
Household income			
Average or above	1.0		
Below average	1.42	0.83–2.42	0.21
Illness severity			
No/minor	1.0		
Moderate	2.40 **	1.38–4.20	0.002
Severe	6.59 ***	3.43–12.69	<0.0001
Experienced stigma			
Low	1.0		
High	2.24 **	1.36–3.67	0.001

OR: odds ratio; CI: Confidence Interval. ** *p* < 0.01, *** *p* < 0.001.

## Appendix

**Stigma Questionnaire**

1 The local residents are unwilling to chat or have a talk with TB patients.
2 Many residents keep their distance from TB patients.
3 The neighbors will treat the TB patients differently once they get the news.
4 Many people walk away from facing TB patients.
5 My family members feel that they have lost face after knowing my illness with TB.
6 Local residents will not let their kids come close to TB patients.
7 People will not have dinner together with relatives or friends of TB patients.
8 TB patients are not welcome by their local villagers or community residents.
9 I think it is no good to inform the others of my TB infection.

(Strongly disagree = 1, disagree = 2, agree = 3, strongly agree = 4)
